# Heat-induced symmetry breaking in ant (Hymenoptera: Formicidae) escape behavior

**DOI:** 10.1371/journal.pone.0173642

**Published:** 2017-03-29

**Authors:** Yuan-Kai Chung, Chung-Chi Lin

**Affiliations:** 1 Department of Physics, National Chung-Hsing University, Taichung, Taiwan; 2 Aqua Fairy Biotechnology Company Limited, Taichung, Taiwan; 3 Department of Biology, National Changhua University of Education, Changhua, Taiwan; Waseda University, JAPAN

## Abstract

The collective egress of social insects is important in dangerous situations such as natural disasters or enemy attacks. Some studies have described the phenomenon of symmetry breaking in ants, with two exits induced by a repellent. However, whether symmetry breaking occurs under high temperature conditions, which are a common abiotic stress, remains unknown. In our study, we deposited a group of *Polyrhachis dives* ants on a heated platform and counted the number of escaping ants with two identical exits. We discovered that ants asymmetrically escaped through two exits when the temperature of the heated platform was >32.75°C. The degree of asymmetry increased linearly with the temperature of the platform. Furthermore, the higher the temperature of heated platform was, the more ants escaped from the heated platform. However, the number of escaping ants decreased for 3 min when the temperature was higher than the critical thermal limit (39.46°C), which is the threshold for ants to endure high temperature without a loss of performance. Moreover, the ants tended to form small groups to escape from the thermal stress. A preparatory formation of ant grouping was observed before they reached the exit, indicating that the ants actively clustered rather than accidentally gathered at the exits to escape. We suggest that a combination of individual and grouping ants may help to optimize the likelihood of survival during evacuation.

## Introduction

One of the most fascinating phenomena predicted by Helbing et al. is the asymmetry in escaping observed when humans escaped a room with two identical exits in an emergency situation [1]. Helbing et al. proposed a model based on physical and sociopsychological forces to describe human crowd behavior in panic situations. To achieve optimal benefits (lowest casualties) in an evacuation system, humans would utilize two symmetric choices equally. However, panic-induced symmetry breaking occurred when humans escaped from a room with two exits. Individuals instinctively followed the crowd instead of rationally choosing the less crowded exit, resulting in chaos and jamming at one exit [2, 3]. The phenomenon of collective asymmetry in escaping also occurs in ants.

Altshuler et al. used citronella oil as a repellent to create a panic condition for leaf-cutting ants (*Atta insularis*) and observed asymmetry in the numbers of escaping ants through two exits [4]. In addition, ants tended to escape in groups rather than as individuals [5]. In these studies, the panic condition was created by citronella oil, which is not a widespread hazard in the wild. Few studies have investigated the escape behavior of ants that used two exits when encountering natural abiotic threats such as heat.

Ants are poikilothermic social insects, which are sensitive to extreme abiotic conditions such as high temperatures. In a thermal stress environment, ants are not able to forage or survive [6, 7]. Heat from the sun or an artificial source rapidly elevates the body temperature of these small-bodied poikilotherms to lethal levels. Ants cannot forage at the critical thermal maxima, defined as the temperature limit for egress from a lethal environment [8]. They respond behaviorally and physiologically to circumvent or minimize potential injury. For instance, ants retreat to a moderate environment, such as by resting in the shade, to avoid the risk of mortality caused by heat [6, 9]. Representative ant survival strategies (e.g., changing response patterns or increasing ant foraging activity) are the first form of defense against high-temperature induced injury [10]. Both the ambient temperature and the surrounding environment determine their survival strategies; however, how they escape from a narrow space with two identical exits under thermally induced danger remains unknown.

Our study focused on the escape behavior of ants from a space with two identical exits under high temperature. Experiments were conducted using a heating platform at temperatures ranging from 22 to 45°C. After integrating our findings with those of other studies [4, 5, 11], we suggest that the collective asymmetry in escaping under different panic (heating or repellent) conditions is a general behavior in ant species.

## Materials and methods

### Ethics statement

We did not involve endangered or protected species in this study. All efforts were made to minimize suffering in experiments.

### Ants

The black spiny weaver ant, *Polyrhachis dives* (F. Smith), is an arboreal ant, which is mainly located in the low-lying areas of Taiwan. Its nests are usually found in weeds or shrubs. Six colonies were collected from Nantou county, Taiwan, and transferred to our laboratory. There is no specific permissions were required for these locations/activities. Each colony contained 1500–2500 workers and dozens of queens. Ants were maintained in a plastic box with a vial of distilled water and food. The internal walls of the containers were brushed with Fluon (Northern Products Inc., Rhode Island, USA.) to prevent the ants from escaping. The colonies were kept at approximately 25°C, under a natural light–dark cycle.

### Experimental device

**Fig 1:**
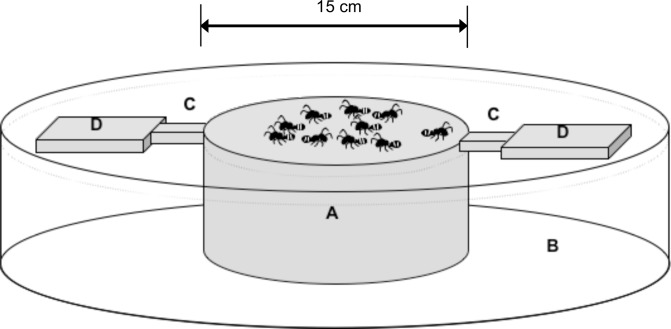
Schematic diagram of the experimental setup. (A) heated platform, (B) thermostatic water bath, (C) thermal insulating bridges, and (D) unheated platform.

We built a device (Fig 1) consisting of a heated metal platform with two opposite bridges connecting to an unheated platform. The heated iron platform (Fig 1A) was 15 cm in diameter, which was sufficient for evenly distributing 3000 black spiny weaver ants without their touching each other. Most of the heated cylinder, except for the heated platform, was immersed in a thermostatic water bath (Fig 1B), and the temperature of the heated platform could be controlled through the thermostatic water bath. The two opposite bridges acting as exits (Fig 1C) were 3 cm in width and were connected to the heated and unheated platforms (Fig 1D). The bridges were made of acrylic, a thermal insulating material, to prevent heat conduction from heated platform.

### Experimental procedure

We investigated whether asymmetry in escaping through two identical exits occurred under thermal stress at different temperatures (22, 25, 30, 35, 36, 37, 38, 39, 40, 41, 42, 43, 44, and 45°C). The thermostatic water bath was operated for a while before the start of each experiment to enable the heated platform reach the target temperature. In each experiment, 60 ant workers were haphazardly selected from the one of the artificial nests in the laboratory and placed in a plastic cup. The internal wall of the cup was brushed with Fluon to prevent the ants from escaping. The ants in the cup were then simultaneously placed on a heated platform. Once the ants were deposited on the heated platform, they could escape. The escape process was recorded for 3 min through a video camera set up above the arena. The number of escaping ants whose bodies were completely passed through each exit (bridge) was counted. And the ants were immediately removed from exits to prevent them from returning to heated platform. Twenty replicates were conducted at each temperature. Ants for evacuation experiments were from the same artificial nest in laboratory and different ant workers were used for each experiment. The entire device was covered with cloth to minimize the interference of surrounding environments with the ants during the experiment.

### Statistical analysis

The relative difference in the use of two exits was calculated at each temperature using the following formula from Altshuler et al. (2005) [4]. Assuming that each ant escaped independently, the use of two exits would be nearly equal. The chi-square goodness-of-fit hypothesis test was used to determine the asymmetry in escaping. We also used non-linear regression to identify the temperature threshold at which symmetry breaking in the escaping ants was induced.

$$\frac{\left|\mathrm{escaping ants using left exit}–\mathrm{escaping ants using right exit}\right|}{\mathrm{total escaping ants}}\times 100$$

To examine whether the ants preferred collective motion under high temperatures, we defined more than three ants moving collectively as a group, in which the distance between each ant was less than one ant length (6 mm). The relationship between the proportion of ant grouping at the exits and different temperatures was examined using regression analysis. All statistics were calculated using Statistica 7 software (Dell Inc., Texas, USA).

## Results

### Symmetry breaking induced under high temperature

**Fig 2:**
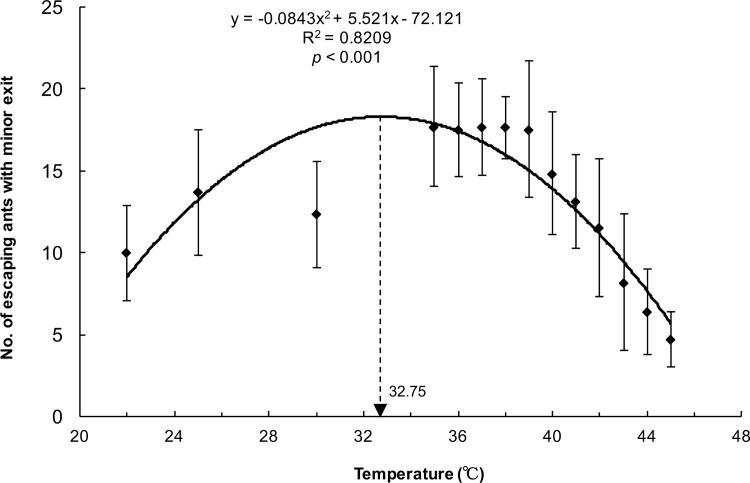
Number of ants escaping through the minor exit at different temperatures in 3 min. The relationship between the ants escaping through the minor exit and the platform temperature was significant in the polynomial regression analysis.

**Fig 3:**
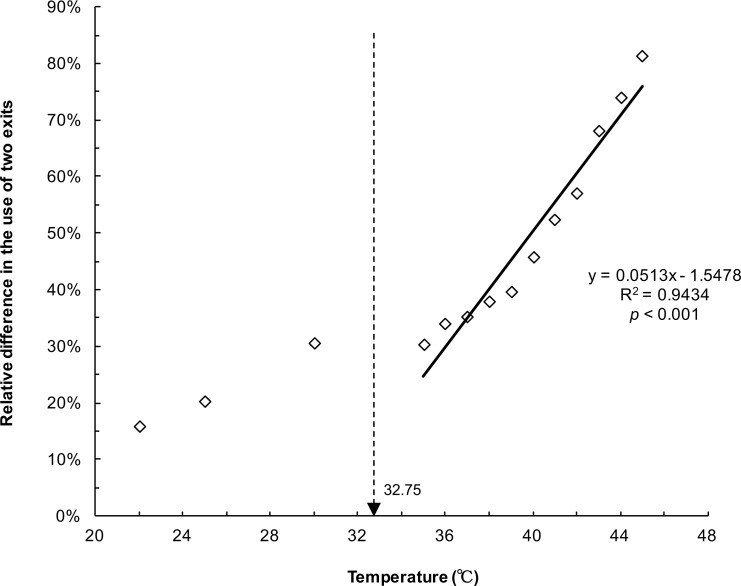
Relative difference in the use of the two exits at different temperatures in 3 min. The regression line corresponds to the relationship between the relative difference in the use of the two exits and the ground temperature.

Fig 2 demonstrates the number of ants escaping from the minor exit at different heated platform temperatures (22, 25, 30, 35, 36, 37, 38, 39, 40, 41, 42, 43, 44, and 45°C), assessed through polynomial regression analysis (y = −0.0843x^2^ + 5.521x − 72.121, *R*^2^ = 0.8209, *p* < 0.001). The peak of the regression curve was at 32.75°C. This indicated the critical temperature at which a turning point from symmetrical to asymmetrical egress occurs with two exits. Under this critical temperature, no significant relative difference (χ^2^ < 3.21, *p* > 0.073) was observed in the use of two identical exits (Table 1). When the temperature was higher than 32.75°C (from 35 to 45°C), symmetry breaking occurred (χ^2^ > 4.49, *p* < 0.05). The relative difference in use of the two exits was lowest (15.6% ± 12.8%) at 22°C, whereas it increased up to 81.1% ± 6.2% at 45°C. Symmetry breaking in the escaping ants was triggered by heat signals from the heated platform. In addition, the relative difference in the use of the two exits increased monotonically with the rise in temperature (y = 0.0513x − 1.5478, R^2^ = 0.9434, *p* < 0.001; Fig 3), implying that the degree of asymmetry in escaping reflected the agitation level of the ants under heat stress.

**Table 1:** Number of escaping ants and relative difference between the two exits at different temperatures in 3 min.

Temp. (℃)	No. of major exit (SD)	No. of minor exit (SD)	Total No. escaping ants (SD)	Relative difference of two exits (SD)	χ^2^	*p*-value
22	13.4 (3.1)	10.0 (2.9)	23.4 (5.4)	15.6 (12.8)	0.49	0.486
25	20.4 (4.0)	13.7 (3.8)	34.1 (7.3)	20.1 (9.1)	1.30	0.256
30	23.0 (3.6)	12.4 (3.2)	35.4 (4.1)	30.4 (16.4)	3.21	0.073
35	32.8 (2.4)	17.7 (3.7)	50.5 (2.3)	30.2 (12.6)	4.49	0.034
36	35.3 (2.6)	17.5 (2.8)	52.8 (2.6)	33.8 (9.6)	6.00	0.014
37	36.9 (2.9)	17.7 (3.0)	54.6 (2.1)	35.2 (10.2)	6.72	0.010
38	39.1 (1.3)	17.7 (1.9)	56.7 (1.6)	37.8 (5.5)	8.08	0.004
39	40.1 (2.9)	17.6 (4.2)	57.6 (3.8)	39.4 (11.3)	8.79	0.003
40	39.6 (3.4)	14.9 (3.7)	54.5 (2.0)	45.6 (12.9)	11.25	0.001
41	41.7 (2.3)	13.2 (2.8)	54.9 (1.4)	52.2 (9.5)	14.86	< 0.001
42	41.7 (3.6)	11.6 (4.2)	53.3 (2.2)	56.9 (14.7)	17.07	< 0.001
43	42.4 (3.9)	8.2 (4.2)	50.6 (3.3)	67.9 (15.0)	23.12	< 0.001
44	42.4 (3.0)	6.4 (2.6)	48.8 (2.7)	73.9 (10.3)	26.56	< 0.001
45	44.7 (2.1)	4.7 (1.7)	49.4 (2.7)	81.1 (6.2)	32.39	< 0.001

Asymmetry in escaping was determined using the chi-square goodness-of-fit test.

### Number of escaping ants was influenced by temperature

**Fig 4:**
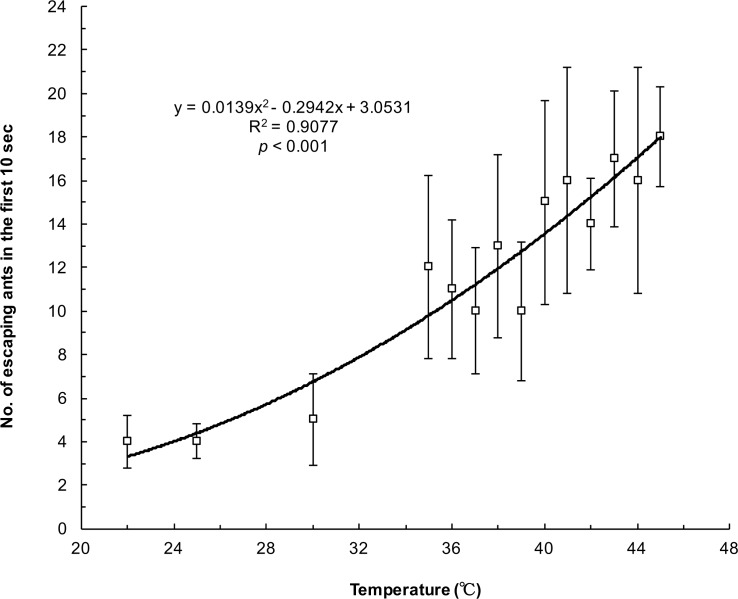
Number of escaping ants in the first 10 s at different temperatures. The curve represents the relationship between the number of ants escaping in 10 s and the heated platform temperature, determined through regression analysis.

**Fig 5:**
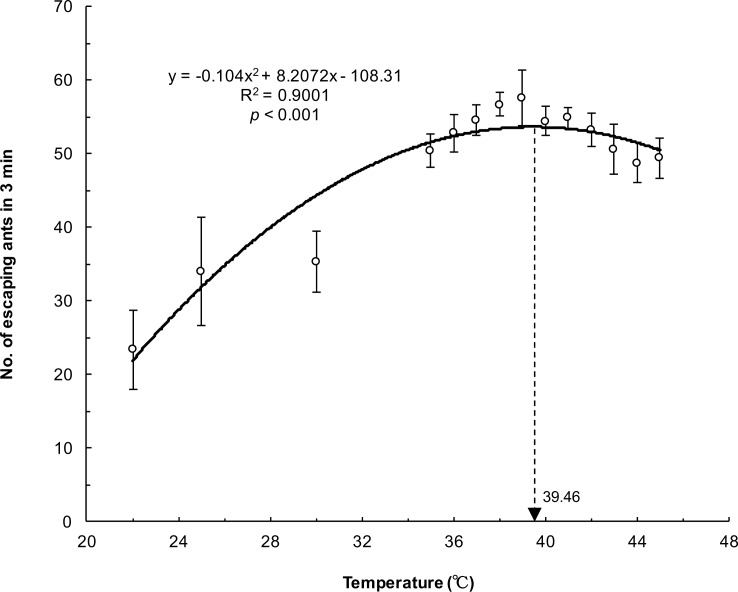
Number of escaping ants in 3 min at different temperatures. The relationship between escaping ants and platform temperatures was significant as per polynomial regression analysis.

Fig 4 depicts the number of escaping ants in 10 s under different heated platform temperatures. The number of escaping ants gradually increased in conjunction with the temperature (y = 0.0139x^2^ − 0.2942x + 3.0531, R^2^ = 0.9077, *p* < 0.001). At the highest temperature (45°C), more than 25% of ants could efficiently escape in merely 10 s. The evacuation speed of the ants was indirectly regulated by the environment temperature. However, the maximum number of escaping ants was not observed at the highest temperature (45°C) in the 3 min period (Table 1). Non-linear regression showed that the peak of the regression curve was at 39.46°C for the 3 min period (Fig 5; y = −0.104x^2^ + 8.2072x − 108.31, *R*^2^ = 0.9001, *p* < 0.001), indicating the critical thermal maxima. The number of ants escaping in 3 min increased when the temperature rose, whereas it began to decline when the temperature was >39.46°C. The trends in these two results (the number of ants escaping within 10 s and 3 min) were converse when the temperature was >40°C.

### Collective motion of ants under thermal stress

**Fig 6:**
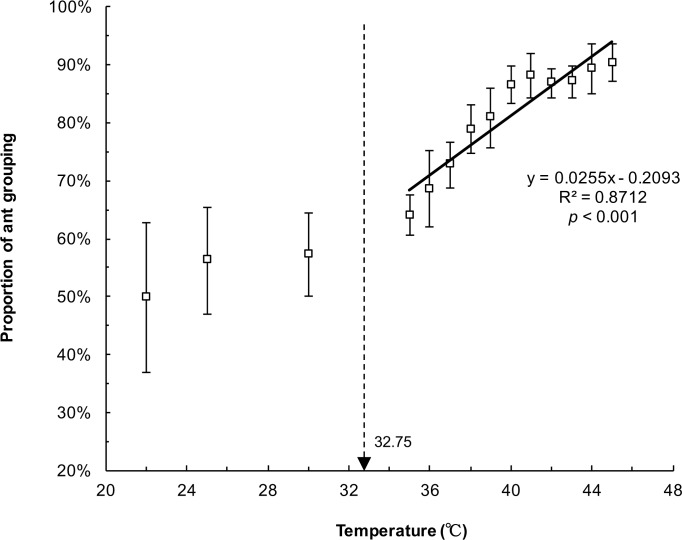
Proportion of ants grouping to escape in 3 min at different temperatures. The line shows the relationship between the percentage of ant grouping and heated platform temperature.

**Fig 7:**
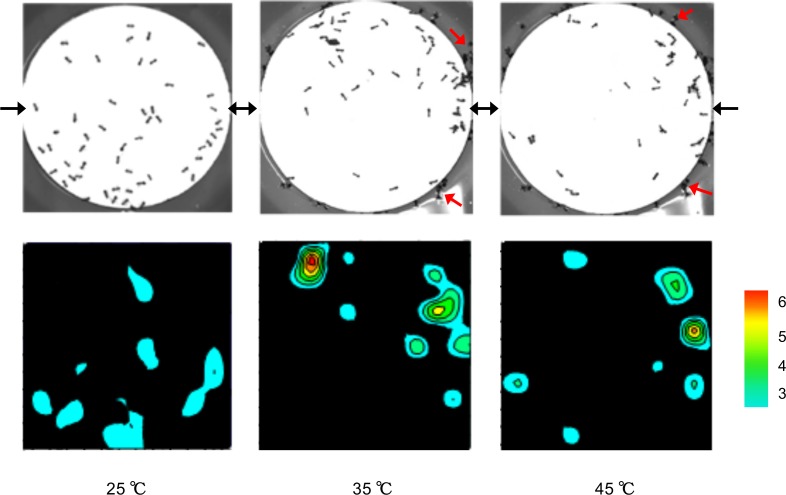
Snapshots of ant distributions on the heated platform at different temperatures at 15 s. The color indicates the ant numbers in clusters. The black arrows indicate the location of the exits (bridges) on the heated platform. The red arrows indicate that the ants fell from the heated platform and floated on the surface of water.

Fig 6 demonstrates the proportion of ant groups escaping through the exits at different temperatures. Linear regression analysis showed a significant relationship between collective escaping and heated platform temperatures (y = 0.0255x − 0.2093, R^2^ = 0.8712, *p* < 0.001). The proportion of ant grouping grew linearly when the temperature (> 32.75°C) increased. Ants preferred escaping in groups (more than three ants per cluster) instead of as individuals under thermal stress. At the lowest temperature, the proportion of ants escaping in groups was similar to that of ants escaping individually. At the highest temperature (45°C), the amount of ant groups was nine-fold higher than that of the individuals. Furthermore, the distribution of ants on the heated platform was examined at 15 s to investigate whether the escaping ants tended to cluster before they reached the exit. Some of the ants formed small groups (3–6 ants) at 25°C, whereas they clustered more closely at 35 and 45°C (Fig 7). This implied that they actively clustered into piles initially and then escaped in groups when they encountered thermal stress. Although we also observed that some ants fell from the heated platform and floated on the surface of water (Fig 7), it didn’t influence our consequence. Because there were still a majority of ants (over than 80%) escaping without falling or dying under heat stress (over 35°C) (Table 1).

## Discussion

In our study, an asymmetry in the escaping of *P*. *dives* ants from two identical exits was induced under high ground temperatures (>32.75°C; Fig 2), which are a common abiotic danger for ants. Other researchers have also observed symmetry breaking in different ant species, including *Camponotus japonicas* [12], *Atta insularis* [4], and *Solenopsis invicta* [11]. This phenomenon may not be species-dependent in ants. Some studies have continued exploring the mechanism responsible for the asymmetry in escaping. So far, a more acceptable explanation is that escaping ants recognize the major exit through pheromones, which are a chemical signal used to communicate among ants. Many ant species have poor vision, so they leave trail pheromones as cues for other members to track [13]. Other ants can perceive these pheromones through their antennae and can determine whether to follow [14]. Li et al. (2014) also proposed an alarm pheromone model to explain the effect of symmetry breaking on ant density [11]. They inferred that pioneer ants randomly chose one exit and deposited a pheromone along the way; the rest of the ants sensed it and followed subsequently.

Helbing et al. (2000) proposed the “faster is slower” (FIS) effect among pedestrians during room evacuation through computer simulation [1]. When individuals tried to escape in shortest time by following a straight path to the exit, clogging and jamming occurred at the exit. This model corresponded to the crowded situation observed at the exit during the “Station Nightclub” fire (USA, 2003) [15]. The mechanism of the FIS effect may involve the tangential friction of contacted particles [16]. In addition, Soria et al. identified the FIS phenomenon in escaping ants [17]. The evacuation time decreased as the concentration of citronella oil increased. However, the evacuation time started to increase beyond 75% citronella oil use. The mechanism underlying the FIS effect in ants differed from that in humans because jamming or clogging at the exit was not observed in ants. Boari et al. (2013) mentioned that the movement and performance of ants were probably damaged by the high concentration of citronella oil [18]. Our results also documented the FIS phenomenon in the number of escaping ants and heated platform temperatures (Fig 5). When the temperature was higher than the critical thermal limit (39.46°C) for *P*. *dives*, the locomotion might have been impaired. Therefore, the number of escaping ants began to decline. Interestingly, the number of ants escaping in 10 s monotonically increased, even >39.46°C (Fig 4). We surmised that the performance of the ants was not hampered within 10 s. Soon after, their locomotion was gradually impaired by thermal ground temperatures. Eventually, some of the ants could not escape after 3 min, and they even died.

The ants efficiently evacuated with the degree of thermal stress (Fig 4). This result was similar with that in Boari et al. (2013) study [18]. Other researchers investigated different factors affected the ants’ flow rate through the exit during evacuation. Wang et al. (2015) placed the *C*. *japonicus* ants in single-exit room with repellent and found that the mean flow rate didn’t linearly increased with the exit width [5]. Their group also conducted another experiment to examine whether the distance between two exits in a room influenced the ants’ egress flow rate [12]. They demonstrated that the maximum mean flow rate occurred when the distance was longest (6 cm) compared with other separations between two exits (1 cm, 2.5 cm, 4 cm, and 5 cm). Furthermore, they looked into the relationship between flow rate and density using small and large size of *C*. *japonicus* ants in unidirectional passageway under stress condition [19]. They revealed that the flow rate had significantly positive correlation with the density in both small and large size ants. These studies provided important information to understand the difference of egress behavior between ants and human.

Ants are one of eusocial insects and the priority of survival is colony instead of individual. They prefer collective action to accomplish tasks such as foraging, defense against enemies, and nest moving [20–23]. In our study, the majority of the ants escaped in groups through the major exit under thermal stress, thereby achieving colony movement (Fig 6). They formed groups on the heated platform in advance and escaped through the exits in these groups (Fig 7), implying that ant grouping was not a coincidental gathering at the exit but an actively cooperative behavior. This grouping behavior was also mentioned by Wang et al. (2015) [5]. Moreover, Helbing et al. (2002) proposed the “ignorance of available exits” model, which suggested that neither simple individualistic nor herding behavior is optimal for escaping [24]. Pure individual behavior means that each person just fortuitously finds an exit. Pure herding behavior infers that the whole crowd eventually moves in the same direction while other available exits are not efficiently used. Therefore, they expected that a combination of individualistic and herding behavior can achieve optimal chances of survival. This model may provide a rational interpretation of our findings. Individual ants were allowed to find the exit, and grouping ants that successfully identified the solution were imitated by small groups. Both small-group formation and a certain proportion of individuals were essential for ants to escape through the available exits under panic conditions.

## Conclusions

The Chinese proverb “like an ant on a hot pot” has a similar meaning to the English proverb “like a cat on a hot tin roof.” However, the escape behavior of ants from a space with two identical exits under “hot pot” conditions was not clear. We investigated whether an asymmetry in the escaping of *P*. *dives* ants was induced on a heated metal platform. When the ground temperature grew to >32.75°C, the relative difference in the use of the two exits significantly increased. The FIS phenomenon was observed in the number of escaping ants and ground temperature in 3 min. However, the mechanism underlying the FIS effect differed between humans and ants.

In addition, ant grouping was noticed at the exits under thermal stress. Some of the ants had already formed groups on the heated platform, even before they crossed the exits. We speculated that a mixture of individual and grouping ants may help in achieving the highest possibility of survival during evacuation.

Our study provided evidence that ants exhibit collective asymmetry in escaping from two identical exits under heat stress. Consistent with previous findings, this behavior commonly occurs in different ant species under panic conditions.
